# Neuroprotection in Early Diabetic Retinal Disease Using Eyedrop Delivery

**DOI:** 10.3390/ijms27125553

**Published:** 2026-06-19

**Authors:** Hugo Ramos, Olga Simó-Servat, Cristina Hernández, Rafael Simó

**Affiliations:** 1 Diabetes and Metabolism Research Unit, Vall d’Hebron Research Institute (VHIR), 08035 Barcelona, Spain; olga.simo@vallhebron.cat (O.S.-S.); cristina.hernandez@vhir.org (C.H.); 2 Center for Networked Biomedical Research of Diabetes and Associated Metabolic Diseases (CIBERDEM), Carlos III Health Institute (ICSIII), 28029 Madrid, Spain; 3 Department of Medicine, Autonomous University of Barcelona, 08193 Barcelona, Spain

**Keywords:** diabetic retinal disease, diabetic retinopathy, neurodegeneration, retina, neuroprotection, eyedrops, transscleral, glaucoma

## Abstract

Diabetic retinal disease (DRD) has classically been defined as a microvascular complication of diabetes; however, the recent evidence highlighted the key role of neuronal degeneration during the earliest stages of its pathogenesis. Therefore, neuroprotection has emerged as a promising therapeutic strategy to prevent disease progression. Topical administration via eyedrops represents a non-invasive approach to deliver neuroprotective agents directly to the retina. This review summarizes the current advances in the field of neuroprotective therapies against early DRD with a special focus on topical delivery, including preclinical and clinical evidence, while discussing the relevance of the transscleral route of absorption in all of them. In this review, the most promising neuroprotective compounds under development will be discussed, highlighting the opportunity that they represent for treating early stages of DRD.

## 1. Introduction

Diabetic retinopathy (DR) is one of the leading causes of visual impairment worldwide and has classically been defined as a microvascular pathology; however, recent evidence demonstrated that retinal neurodegeneration is an early and critical component of the pathophysiology of this complication of diabetes [1,2,3]. In fact, loss of retinal ganglion cells and alterations in synaptic transmission may occur even before vascular lesions become clinically evident [4,5,6,7]. To better capture the full spectrum of retinal alterations induced by diabetes, including both neurodegenerative and microvascular components, the term diabetic retinal disease (DRD) has been proposed as a broader definition, whereas classical DR has traditionally been regarded primarily as a microvascular pathology [2].

Current treatments, such as laser photocoagulation, intravitreal injections of anti-vascular endothelial growth factor (VEGF) or corticosteroids, target the advanced stages of the disease, when vision is already impaired and are aimed to address vascular abnormalities rather than neurodegeneration [8,9]. Although the effectiveness of these approaches has been proven, they are invasive and have been associated with significant side effects such as endophthalmitis, retinal detachment, intraocular inflammation, cataract formation or glaucoma [10,11,12]. Moreover, long exposure to anti-VEGF therapies has been associated with interferences with the neuroprotective functions of VEGF, worsening retinal neurodegeneration [13,14].

In this context, there is a growing interest in therapeutic strategies that can prevent or arrest retinal neuronal damage during the earliest stages of DRD [15,16]. Since an invasive treatment cannot be justified in these early stages of the disease, increasing attention has been given to non-invasive treatments with minimal adverse effects, such as the case of [17,18] topical ophthalmic approaches (i.e., eyedrops) [19]. Despite previous uncertainty regarding the ability of eyedrop formulations to reach the posterior segment of the eye, emerging evidence has demonstrated that this route represents an efficient non-invasive approach for delivering neuroprotective agents to this region, at least in experimental models [20,21,22].

This narrative review was based on a literature search conducted in PubMed/MEDLINE and Web of Science covering studies published from January 2000 to March 2026. Searches were performed using combinations of keywords related to diabetic retinal disease, diabetic retinopathy, retinal neurodegeneration, neuroprotection, topical ocular therapies, eyedrops, transscleral drug delivery, and retinal biomarkers. Priority was given to peer-reviewed original research articles, clinical studies, and mechanistic investigations considered most relevant to the objectives of this review.

## 2. Retinal Neurodegeneration in Early Diabetic Retinal Disease

Retinal neurodegeneration is initially triggered by chronic hyperglycemia, which through the activation of a complex network of different biochemical mechanisms leads to neuronal damage [23]. One of the principal mechanisms involved is oxidative stress [24]. Sustained high glucose levels over time result in an excessive production of reactive oxygen species (ROS) due to mitochondrial dysfunction and the activation of metabolic pathways such as the polyol pathway, protein kinase C pathway, hexosamine pathway and the formation of advanced glycation end-products (AGEs) [25,26]. The accumulation of ROS damages key cellular components, including lipids, proteins, and DNA/RNA, leading to neuronal dysfunction and apoptosis [27]. It is important to mention that retinal neurons are particularly vulnerable to oxidative stress due to their high metabolic demand and limited antioxidant capacity [28].

All these detrimental effects impair the normal functionality of the retinal neurovascular unit (NVU), which consists of retinal neurons, glial cells, and vascular cells, and plays a key role in maintaining retinal homeostasis. One of the main functions is to ensure the so-called neurovascular coupling, which refers to the blood flow regulation in response to neuronal metabolic demands. In early DRD, disruption of the NVU leads to impaired neurovascular coupling, resulting in inadequate oxygen and nutrient delivery and contributing to both neuronal and vascular dysfunction [29,30,31].

Alterations in glial cell function represent another important component of DRD which is involved in the early neurodegenerative process [32,33,34]. Müller cells are responsible for providing metabolic and structural support to retinal neurons, regulating ion and neurotransmitter homeostasis, and preserving retinal integrity [35,36]. In diabetes, these cells undergo reactive gliosis or glial activation, which is characterized by upregulation of glial fibrillary acidic protein (GFAP), impaired potassium and glutamate levels, and altered secretion of neurotrophic factors. The loss of this supportive role contributes to neuronal vulnerability and progressive neural degeneration [35,37,38]. In addition, glial activation is associated with an overexpression of VEGF and proinflammatory cytokines that play a key role in provoking early impairment of capillary permeability and loss of blood-retinal barrier (BRB) sealing, thus resulting in vascular leakage [32,37,39,40].

Glutamate excitotoxicity is a key mechanism implicated in retinal damage in the setting of DRD [41,42]. Under physiological conditions, glutamate is released during synaptic transmission and is regulated by Müller glial cells, which are responsible for controlling their extracellular levels. In DRD, Müller cell dysfunction impairs glutamate uptake, causing its accumulation in the extracellular space. The abnormal increased levels of glutamate overstimulate the ionotropic glutamate receptors, particularly N-methyl-D-aspartate (NMDA) receptors, resulting in excessive calcium influx into neurons [43,44,45]. This intracellular calcium overload activates enzymatic pathways that promote mitochondrial damage, oxidative stress, and ultimately neuronal cell death [46].

In addition to oxidative stress, nitrosative stress has emerged as an important contributor to retinal neurodegeneration in DRD. Excessive activation of NMDA receptors promotes intracellular calcium accumulation, which stimulates neuronal nitric oxide synthase (nNOS) activity and increases the production of nitric oxide (NO). Simultaneously, chronic hyperglycemia and inflammation up-regulate the expression of inducible nitric oxide synthase (iNOS) in retinal neurons and glial cells. Excessive NO reacts with superoxide radicals to generate peroxynitrite and other reactive nitrogen species, resulting in protein nitration, mitochondrial dysfunction, DNA damage, and amplification of inflammatory signaling pathways [47,48,49,50]. Consequently, nitrosative stress acts as an important mechanistic link between excitotoxicity, oxidative stress, and neuronal injury in DRD [48].

Inflammation plays a crucial role not only in the development of vascular leakage, but also in retinal neurodegeneration [39,51,52]. Chronic hyperglycemia activates retinal microglia [53], which adopt a pro-inflammatory phenotype and release cytokines such as tumor necrosis factor-alpha (TNF-α) or interleukin-1β (IL-1β) [54]. These inflammatory mediators exacerbate the neuronal damage by increasing oxidative stress, disrupting the synaptic machinery, and activating pro-apoptotic pathways. In addition, chronic inflammation also aggravates the dysfunction of Müller cells, further amplifying the neurodegenerative process [39,55].

Besides classical apoptosis, increasing evidence indicates that other regulated cell-death pathways contribute to retinal neurodegeneration in DRD [56,57,58]. In particular, pyroptosis, an inflammatory form of programmed cell death mediated by inflammasome activation, caspase-1 cleavage, and gasdermin-dependent membrane pore formation, has emerged as a relevant mechanism. Pyroptosis promotes the release of pro-inflammatory cytokines such as IL-1β and IL-18, thereby amplifying retinal inflammation and neuronal damage. Importantly, apoptosis and pyroptosis are not mutually exclusive processes and may coexist during disease progression, contributing to neuronal loss, glial dysfunction, and disruption of retinal homeostasis [56,57,58,59].

Altogether, these mechanisms provoke structural and functional alterations in all retinal cell types, including retinal ganglion cells, amacrine cells, and photoreceptors [3,60]. Progressive neuronal loss and synaptic dysfunction contribute to early visual deficits observed in diabetic patients, even before the development of overt microvascular lesions. These findings highlight the importance of targeting neurodegenerative pathways as a potential strategy to prevent or delay the progression of DRD [15,16,61].

## 3. Neuroprotective Strategies Targeting Early Diabetic Retinal Disease

Based on the current evidence of pathophysiological mechanisms that trigger neuronal degeneration in DRD, several pharmacological approaches have been proposed to counteract them [16,62]. First, antioxidant therapies may reduce oxidative stress and mitochondrial damage [63,64], while anti-inflammatory agents have the potential to attenuate chronic retinal inflammation and microglial activation [65,66]. There are also experimental strategies designed to modulate glutamate signaling to prevent excitotoxicity [67], as well as approaches that use neurotrophic or neuromodulatory factors that promote neuronal survival and maintain synaptic function [21,68,69,70,71,72,73]. By acting on these mechanisms, neuroprotective therapies could potentially slow or prevent the progressive neuronal loss that characterizes the early stages of DRD.

Among these neuroprotective therapies, those aimed at replacing the loss of neurotrophic factors in the retina due to diabetes are particularly noteworthy. Retinal production of neuroprotective factors such as pigment epithelium-derived factor (PEDF), somatostatin (SST), Interphotoreceptor retinol-binding protein (IRBP) and glucagon-like peptide-1 (GLP-1) are downregulated in the diabetic retina and, therefore, their replacement can be contemplated as a rationale treatment to prevent or arrest retinal neurodegeneration [21,74,75,76,77,78,79].

### 3.1. Neuroprotection Using Topical Administration

Among the different therapeutic approaches under investigation, systemic long-term administration of neuroprotective agents presents several important limitations. These include the difficulty in achieving therapeutic concentrations in the retina due to the inability of some compounds to cross the BRB [17,80,81], potential systemic toxicity and the risk of pharmacological interactions in patients who are frequently poly-medicated, as occurs in diabetes [82,83].

In this context, treatments administered through ophthalmic eyedrops have gained more attention during the most recent years. Topical administration represents an especially attractive strategy due to its non-invasive nature and favorable safety profile compared with current treatments that use invasive intravitreal injections [9]. In addition, the reported effectiveness has been very high, at least in experimental models [84]. Furthermore, in the clinical area, the topical route allows self-administration of the drug and reduces visit frequentation and medical interventions due to side effects, thus minimizing the associated costs. However, errors in self-administration of eyedrops may occur, increasing the risk of therapeutic failure [85]. Despite this, with proper patient education in long-term administration of eyedrops [85], maximum benefit of the compounds can be gained by using this route of administration. These characteristics are particularly relevant for chronic diseases such as DRD, where early intervention and long-term treatment may be required. Topical ocular drug delivery is also associated with other well-recognized limitations that can impact its ability to achieve therapeutic levels in the retina. These include (i) the inherently low ocular bioavailability of topical formulations due to tear turnover, blinking, corneal barriers, and nasolacrimal drainage; (ii) translational limitations related to interspecies differences in ocular anatomy, including scleral thickness, ocular surface area, and globe dimensions between animals and humans; (iii) formulation challenges such as enzymatic degradation and drug stability; (iv) practical concerns regarding dosing frequency, adherence, and self-administration in chronic asymptomatic disease; (v) potential local ocular toxicity associated with long-term administration; and (vi) the difficulty of demonstrating therapeutically meaningful retinal concentrations in humans [86,87,88].

#### 3.1.1. Current Experimental Approaches Based on Neuroprotection

Several compounds have been investigated as potential topical neuroprotective therapies [16,89]. These include small molecules, neuropeptides, metabolic modulators, anti-inflammatory drugs, and neurotrophic factors [72,73,74,90,91]. As previously mentioned, recent advances in drug delivery technologies have enabled the development of novel eyedrop formulations designed to enhance ocular penetration and retinal bioavailability. Current approaches include nanoparticle-based carriers, engineered peptide-derived formulations, cell-penetrating peptides, and other advanced delivery systems capable of improving drug stability and facilitating transport to posterior ocular tissues [62,90]. These strategies share the common objective of overcoming ocular barriers that limit drug bioavailability at the retinal level. Nanoparticle-based carriers protect compounds from premature degradation, extend ocular surface retention and promote sustained release, which increases retinal exposure. Likewise, peptide-derived therapeutics, particularly cell-penetrating peptides, may enhance transscleral diffusion and intracellular transport. Overall, these advanced delivery systems are designed to improve drug stability, bioavailability and retinal penetration to posterior ocular tissues [62,90].

Despite only some of the topical therapies having progressed to clinical evaluation, a growing body of preclinical evidence obtained in experimental models of retinal degeneration, DRD, glaucoma, and optic nerve injury suggests that several molecules administered as eyedrops can exert neuroprotective effects at the retinal level [16,89]. The main experimental and clinical approaches using ophthalmic eyedrops for retinal neuroprotection, highlighting their mechanisms of action and stage of development are summarized in Table 1. Collectively, these experimental therapies can be categorized into several mechanistic groups, including neuromodulatory approaches, metabolic neuroprotective agents, anti-inflammatory and immunomodulatory drugs, as well as antioxidant and mitochondrial-protective compounds.

Among the different neuroprotective strategies currently under development, which are presented in Table 1, some compounds have attracted particular attention due to the promising and robust results obtained at a preclinical level and their consequent potential translational applicability. One of the most extensively investigated approaches is based on SST, an endogenous neuropeptide that is physiologically produced in the retina and whose levels are significantly reduced in patients with diabetes. Experimental studies have demonstrated that the topical administration of SST prevents glial activation, reduces retinal inflammation and attenuates neuronal apoptosis in experimental DRD [78,93]. Additionally, results from the EUROCONDOR clinical trial suggested that SST eyedrops may prevent the worsening of preexisting retinal neurodysfunction [94].

Brimonidine is another relevant compound due to its established clinical use in glaucoma and the neuroprotective properties associated with it [113]. Beyond its intraocular pressure-lowering effect, brimonidine activates α2-adrenergic receptors and promotes anti-apoptotic signaling pathways in retinal ganglion cells. Experimental studies in models of DRD have evidenced significant reductions in neuronal cell death, lower levels of oxidative stress markers and the preservation of the retinal structure after topical administration [90,94]. As SST, brimonidine appears useful in preventing the worsening of preexisting retinal neurodysfunction in the EUROCONDOR clinical trial [91]. Importantly, because brimonidine is already used in ophthalmological practice worldwide, its repositioning for DRD may facilitate its future clinical translation.

Special interest has also emerged around therapies targeting the GLP-1 signaling pathway. GLP-1 receptor agonists (GLP-1RAs) such as exendin-4 and liraglutide have demonstrated potent neuroprotective and anti-inflammatory effects in experimental DRD [21,95,114]. Interestingly, topical administration of these compounds preserves the integrity of the NVU in terms of neuroprotection by reducing glial activation, oxidative stress and neuronal apoptosis while preserving the sealing function of the BRB, thus preventing the diabetes-induced vascular leakage [21,115]. This is important because the results are not mediated by their lowering effect in blood glucose levels [21]. Mechanistically, GLP-1 signaling appears to modulate directly through GLP-1R activation several intracellular pathways involved in cell survival, mitochondrial homeostasis and inflammatory responses [70]. In parallel, topical administration of DPP-4 inhibitors, such as sitagliptin, have shown similar or even better results than GLP-1RAs in terms of both neural and vascular protection [22]. On the basis of transcriptomic analyses, sitagliptin has been shown to activate several neuroprotective mechanisms in the diabetic retina, along with anti-inflammatory, antioxidant, anti-glial activation and neuromodulatory effects [22,71,72,97,98]. Despite GLP-1-related mechanisms being clearly involved in those properties, there exist evidence supporting that GLP-1 receptor-independent mechanisms play a key role [116,117], which suggests that sitagliptin may exert part of its neuroprotective actions through mechanisms that are independent of GLP-1 receptor activation.

Neuropeptide-based therapies have also gained increasing attention. PACAP1-38, a peptide with potent anti-apoptotic and anti-inflammatory properties, has demonstrated protective effects in experimental glaucoma and retinal ischemia models after topical instillation [99,100]. Similarly, PEDF-derived peptides have shown the capacity to promote photoreceptor survival and reduce retinal degeneration [74,118,119]. These approaches are particularly attractive because they mimic endogenous neuroprotective pathways naturally present in the retina [120,121].

Another promising strategy involves the use of neurotrophic factors. Topical administration of NGF has demonstrated beneficial effects not only at the corneal level but also in experimental retinal neurodegeneration [73,75,76]. Likewise, BDNF-based nano-formulations have shown the capacity to preserve retinal ganglion cell survival and synaptic plasticity [69,110]. However, because these molecules are relatively large proteins, achieving efficient retinal penetration remains challenging, which explains the increasing interest in nanocarrier systems and advanced delivery technologies [17,80,81].

Antioxidant and mitochondrial-targeted therapies also represent an important area of investigation. Oxidative stress plays a central role in DRD pathogenesis [24,25], and therefore compounds capable of preserving mitochondrial function may provide substantial neuroprotective benefits. In this regard, coenzyme Q10 combined with vitamin E has shown promising neuroprotective effects in both experimental and exploratory clinical studies [108]. Similarly, nano-formulations containing resveratrol or curcumin have demonstrated anti-inflammatory and antioxidant activity capable of reducing retinal damage in diabetic models [104,105].

Finally, ROCK inhibitors such as ripasudil and fasudil deserve particular mention because of their dual vascular and neuroprotective actions [111,112]. Beyond improving retinal blood flow, these compounds reduce neuroinflammation, oxidative stress and neuronal apoptosis. Since some ROCK inhibitors are already approved for glaucoma treatment, they may represent particularly attractive candidates for repurposing in DRD.

Altogether, these findings illustrate the diversity of neuroprotective strategies currently under investigation and highlight the growing interest in topical therapies capable of targeting multiple pathogenic mechanisms involved in retinal neurodegeneration.

#### 3.1.2. The Transscleral Route

Classically, it was assumed that drugs administered as eyedrops did not reach the posterior pole of the eye at significant concentrations [122,123] because of the presence of corneal barrier, tear turnover, conjunctival clearance, and the perceived inability of compounds to traverse intraocular tissues. This dogma limited topical therapy to anterior segment diseases [80,124]. As a result, retinal diseases such as DRD have traditionally relied on systemic treatment or invasive intravitreal administration [16,125,126,127]. However, recent experimental and clinical evidence suggests that certain molecules administered as eyedrops are capable of reaching the posterior segment of the eye through transscleral, periocular, or uveal pathways, allowing them to exert biological effects at the retinal level.

Nevertheless, not all the compounds can reach the posterior segment through the transscleral route. Compounds with low molecular weight and moderate lipophilicity, such as brimonidine or sitagliptin, have shown better diffusion rates across conjunctival and scleral tissues [128,129]. Adequate aqueous solubility also supports transport through the scleral matrix, while low protein binding may improve bioavailability in the periocular environment [129,130]. On the other hand, large or highly hydrophilic molecules, such as native BDNF, NGF, or erythropoietin, present limited permeability due to restricted diffusion within these barriers [128,131]. Therefore, the physicochemical properties of the topical formulations must be considered when designing them to efficiently reach the posterior segment of the eye.

**Figure 1:**
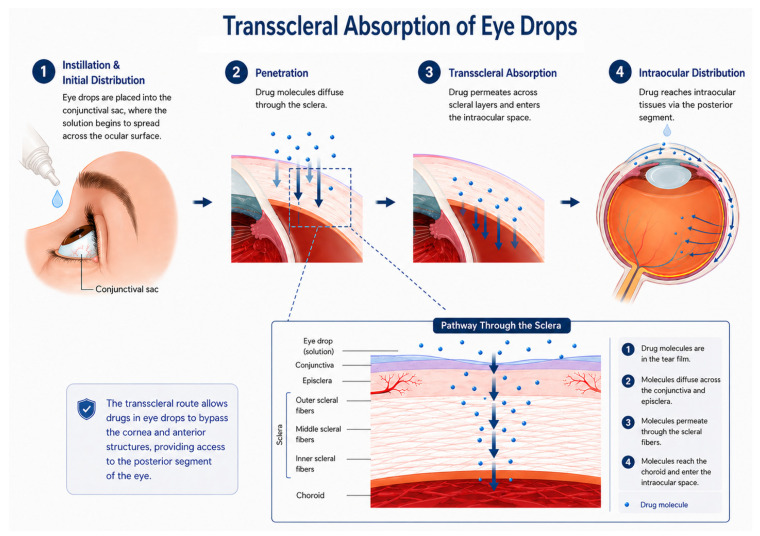
Transscleral route of absorption for topical ocular administration. Topically applied drugs reach the posterior segment via non-corneal routes, mainly through conjunctival and scleral diffusion toward the ciliary body, choroid and retina.

Topical administration of sitagliptin represents a relevant example supporting the feasibility of posterior segment drug delivery via eyedrops [22,132]. Pharmacokinetic data obtained in animal models have shown early and dose-dependent accumulation of the drug in the retina/choroid complex after topical administration, while levels in the aqueous and vitreous humors remain low [132]. This temporal and spatial distribution pattern suggests a predominant transscleral/periocular diffusion pathway (Figure 1) rather than anterior-to-posterior intraocular transport [133,134]. Briefly, this route begins with the distribution of the eyedrops across the tear film, whose formulations are then absorbed through the conjunctiva and penetrate the sclera via diffusion. Once through the sclera, the drug reaches the choroid and, subsequently, the retina, where it exerts therapeutic effects [130,131].

In addition to anatomical accessibility, the pharmacokinetic feasibility of transscleral delivery also depends on whether topically administered compounds achieve and maintain biologically relevant concentrations in posterior ocular tissues. Available evidence suggests that drug exposure following topical administration varies substantially according to physicochemical properties and formulation characteristics. Small molecules with moderate lipophilicity and favorable scleral permeability, such as brimonidine or sitagliptin, appear to exhibit more efficient posterior-segment distribution than larger hydrophilic proteins such as native BDNF, NGF or erythropoietin, whose retinal penetration stills limited without advanced delivery systems [133,135]. In the case of sitagliptin, retinal/choroidal exposure appears rapidly (around 10 min) after topical administration and presents a dose-dependent pattern. Additionally, concentrations detected in aqueous humor and vitreous humor remain comparatively low, supporting a predominant transscleral/periocular route rather than anterior intraocular diffusion [132]. Furthermore, available evidence suggests that repeated dosing may be necessary to maintain sustained tissue exposure over time. Moreover, the pharmacologically active concentrations required for effective neuroprotection remain poorly defined for most compounds under research.

Despite these promising findings, several pharmacokinetic uncertainties remain unclear. Most available evidence derives from animal models, and therefore direct extrapolation to humans should be made with caution. Although current findings support the biological plausibility of topical delivery to the posterior segment, further pharmacokinetic and pharmacodynamic studies are required to better establish the relationship between retinal exposure and therapeutic efficacy, particularly in humans [134,135].

## 4. Biomarkers for Early Detection of Retinal Neurodegeneration in DRD

The successful clinical implementation of topical neuroprotective therapies will depend not only on the availability of effective compounds but also on the identification of reliable biomarkers capable of detecting retinal neurodegeneration at stages in which neuronal damage remains potentially reversible. Furthermore, these biomarkers will be essential for selecting appropriate candidates for treatment and for monitoring therapeutic efficacy in future clinical trials evaluating topical neuroprotective interventions. For this purpose, there are different structural imaging techniques, such as optical coherence tomography (OCT) or OCT angiography (OCTA), that have already been able to detect early changes in the neural components of the retina. In that context, OCT has demonstrated the capability of detecting the thinning of the ganglion cell layer and retinal nerve fiber layer in patients with diabetes before the appearance of the clinically detectable vascular abnormalities [136,137,138], while OCTA has provided evidence of early capillary dropout and reduced vessel density, reinforcing the idea that neuronal and vascular dysfunction coexist from the very beginning of the disease [139,140].

Additional imaging biomarkers associated with retinal neurodegeneration are also emerging. Hyperreflective retinal foci, detected by OCT, are thought to reflect activated microglia and low-grade retinal inflammation and have been associated with the progression of the disease and the disruption of the retinal homeostasis [141,142]. Furthermore, the disorganization of retinal inner layers, initially described in diabetic macular edema, may represent an indirect marker of synaptic dysfunction and neuronal structural impairment [143,144]. Additionally, choroidal parameters, including choroidal thickness and choroidal vascularity index, which are assessed by enhanced-depth imaging OCT, have also been proposed as potential indicators of impaired retinal metabolic regulation and neurovascular dysfunction [145,146].

Besides morphological measurements, functional assessments have revealed even more sensitive in predicting clinical diagnosable signs of DRD [147,148]. Multifocal electroretinography (mfERG), contrast sensitivity testing, dark adaptation studies and visual field assessment have been consistently revealing early neuronal dysfunction in patients with diabetes but without visible funduscopic lesions [149,150,151]. Furthermore, abnormalities in retinal neurovascular coupling measured through flicker-light-induced vasodilation have demonstrated that the physiological vascular response to neuronal stimulation is impaired during the earliest stages of DRD [152]. Retinal oximetry studies have further supported the presence of altered oxygen metabolism and defective vascular autoregulation before clinically detectable microvascular damage becomes evident [153]. Altogether, these findings support the idea of retinal neurodegeneration as an early and independent pathological event instead of merely a secondary consequence of the vascular impairment [2,15].

Beyond imaging and functional assessment, new molecular biomarkers are emerging for the monitoring of disease progression and therapeutic response. In fact, high levels of some inflammatory cytokines, mediators of oxidative stress and extracellular vesicles that carry neuronal or glial signatures have been detected in ocular fluids and plasma from patients with diabetes [39,51,52,54]. Among these biomarkers, TNF-α, IL-1β, VEGF and molecules related to oxidative stress have shown significant associations with retinal dysfunction and the severity of the disease [24,25,51]. Markers of glial activation such as GFAP are also gaining interest due to their close relationship with the impairment of Müller cells and retinal inflammation [32,37].

In addition, the use of biomarkers that are directly related to neuronal injury is also being investigated. Neurofilament light chain (NfL), a well-established biomarker of axonal degeneration in diseases of the central nervous system, has recently emerged as a potential indicator of retinal neurodegeneration [154,155]. In parallel, alterations in synaptic proteins including synaptophysin, PSD-95 and SNAP25 may reflect early synaptic dysfunction preceding irreversible neuronal loss [4,5]. Reduced intraocular levels of endogenous neuroprotective factors such as PEDF, BDNF, NGF, SST and IRBP may also serve as biomarkers of impaired retinal homeostasis and neuronal vulnerability [68,74,75,76,77,78,79,156].

Extracellular vesicles and exosome-associated microRNAs represent another promising field that is rapidly expanding. Retinal neurons, glial cells and vascular cells release extracellular vesicles that contain proteins, lipids and regulatory RNAs which can reflect the metabolic and inflammatory status of the retina [157,158]. Specific microRNAs involved in inflammation, oxidative stress and angiogenesis, including miR-21, miR-126, miR-146a and miR-200b, have been associated with DRD progression and may eventually help with proper patient stratification and prediction of therapeutic response [159,160,161]. These circulating biomarkers are particularly interesting because they could become minimally invasive tools for longitudinal monitoring of retinal neurodegeneration.

Finally, the analysis of retinal images based on artificial intelligence is opening new opportunities for biomarker discovery. Advanced machine learning algorithms applied to OCT, OCTA and fundus imaging may detect subtle structural and vascular abnormalities that are not identifiable by conventional clinical evaluation [162,163]. These approaches have the potential of improving early diagnosis, risk prediction and personalized therapeutic strategies in patients with DRD.

Although additional evidence that confirms the reproducibility and the clinical relevance of these biomarkers is required before their incorporation into the clinical practice their development has the potential of improving the identification of patients with risk of progression and may facilitate the implementation of personalized neuroprotective interventions during the earliest stages of DRD. However, validation in large and independent cohorts, standardization of imaging acquisition and interpretation methods, as well as longitudinal studies demonstrating their ability to predict disease progression and response to neuroprotective interventions are needed.

## 5. Potential Translational Implications of Neuroprotection for Other Retinal Neurodegenerative Diseases: Glaucoma

DRD and other retinal diseases share a core of common neurodegenerative mechanisms. Among them, glaucoma represents one of the most relevant examples because, despite having distinct initiating triggers [chronic hyperglycemia or elevated intraocular pressure (IOP)], both conditions converge on common pathways involving neuroinflammation, oxidative stress, mitochondrial dysfunction, synaptic impairment, and progressive retinal ganglion cell loss [3,164,165]. These shared mechanisms suggest that DRD and glaucoma can be conceptually grouped together in a different framework: chronic neurodegenerative diseases of the retina. Therefore, therapeutic strategies targeting synaptic preservation, redox balance, and inflammatory modulation could have cross-disease applicability. It should also be acknowledged that hereditary susceptibility factors contribute to glaucoma risk and disease heterogeneity, adding further complexity to glaucomatous neurodegeneration and complementing the shared downstream mechanisms discussed above [166].

In this context, a recent study has provided evidence supporting the role of one of the neuroprotective strategies, topical DPP-4 inhibition, in glaucomatous retinal neurodegeneration. In that work, which was conducted in dexamethasone-injected mice, sitagliptin eyedrops preserved RGC survival, reduced glial activation, attenuated pro-inflammatory signaling and improved retinal functionality. Importantly, these effects were independent of systemic metabolic changes, reinforcing a direct retinal mechanism of action [167]. The protective profile described in that study aligns closely with the mechanisms observed in early DRD models treated with sitagliptin eyedrops: inhibition of NF-κB activation, reduction in pro-inflammatory cytokines, attenuation of oxidative stress, preservation of synaptic protein networks, and stabilization of the NVU. The consistency across diabetic and non-diabetic neurodegenerative models suggests that DPP-4 inhibition targets fundamental pathways of retinal homeostasis rather than disease-specific triggers.

From a translational perspective, this is particularly relevant in the context of glaucoma, where most of the current treatment strategies are almost exclusively focused on IOP reduction. A safe topical neuroprotective therapy acting through antioxidant, anti-inflammatory, and synaptic-preserving mechanisms could complement IOP-lowering approaches, especially in patients who continue to progress despite adequate IOP control.

Altogether, the similarities between DRD and glaucoma and the beneficial effects observed in both diseases after topical administration of sitagliptin support the hypothesis that neuroprotective therapeutic strategies may represent a broader neuroprotective strategy applicable to multiple retinal neurodegenerative diseases.

## 6. Conclusions

DRD has recently been recognized and redefined as a neurodegenerative complication, where neurodegeneration has a key role during the earliest stages of the disease. Therefore, there is a need for earlier neuroprotective interventions beyond conventional vascular-targeted therapies designed to arrest the progression of more advanced stages of the disease. Current experimental evidence and preliminary clinical findings suggest that topical administration may offer a feasible and non-invasive approach to deliver neuroprotective compounds to the posterior segment of the eye. However, important challenges remain unsolved, including the need for long-term safety evaluation, validation of sensitive biomarkers for early patient identification and treatment monitoring, and adequately powered randomized clinical trials. Therefore, although topical neuroprotection represents a promising therapeutic approach, further translational and clinical research is required before its potential incorporation into routine clinical practice.

## Figures and Tables

**Table 1:** Experimental neuroprotective therapies delivered as ophthalmic eyedrops for retinal degeneration and DRD.

Therapy/Formulation	Type	Mechanism of Neuroprotection	Disease Model	Dose Regimen	Primary Outcomes Measured	Retinal Drug Levels	Stage
Somatostatin eyedrops [78,92,93,94]	Neuropeptide	Reduces glutamate excitotoxicity and inflammation	Diabetic animal model (STZ-induced diabetes rats and db/db mouse)//Patients with Type II Diabetes	Twice daily	Retinal functionality (mfERG), cell death, glial activation, inflammation, glutamate excitotoxicity	Retinal delivery demonstrated; quantitative retinal levels not reported	Phase II clinical trial
Brimonidine [90,91,94]	α2-adrenergic agonist	Anti-apoptotic signaling in retinal ganglion cells	Diabetic animal model (STZ-induced diabetes rats)//Patients with Type II Diabetes	Twice daily	Retinal functionality (mfERG), Glial activation, cell death//Retinal thickness, level of DR, central retinal arteriolar and venular equivalent	Retinal delivery demonstrated; quantitative retinal levels not reported	Phase II clinical trial/preclinical
GLP-1 receptor agonists (exendin-4, liraglutide) [21,70,95,96]	Metabolic neuroprotection	Anti-inflammatory, antioxidant, anti-apoptotic and neurovascular protection via GLP-1R/Akt/GSK3β signaling	Diabetic animal models (STZ-induced diabetes rats; db/db diabetic mice)	Twice daily	Retinal functionality (ERG), retinal ganglion cell survival, glial activation, oxidative stress, inflammatory cytokines, retinal thickness, tau hyperphosphorylation, apoptosis, neurovascular impairment	Retinal delivery demonstrated; quantitative retinal levels not reported	Preclinical
Sitagliptin eyedrops [71,72,97,98]	DPP-4 inhibitor	Enhances endogenous GLP-1 signaling and other mechanism unrelated to GLP-1R activation	DR and DR-like animal models (db/db mice and TRPV2KO rats)	Twice daily	Retinal functionality (ERG) retinal thickness, cell death glial activation, pro-inflammatory cytokines, levels of synaptic proteins, oxidative damage, levels of antioxidant molecules, diameter of retinal arterioles and venules, acellular capillaries	C_max_: 78.98–134.28 ng/mL in retina/choroid matrix	Preclinical
PACAP1-38 eyedrops [99,100]	Neuroprotective peptide	Anti-apoptotic and anti-inflammatory signaling	Animal model with glaucoma (microbead-induced model)	Three times/day	IOP, retinal morphology, retinal thickness, retinal functionality (ERG), glial activation, cell death	Retinal delivery demonstrated; quantitative retinal levels not reported	Preclinical
PEDF-derived peptide eyedrops (17-mer, H105A) [101]	Neuroprotective peptide	Promotes photoreceptor survival	Murine and human retinal degeneration models (photoreceptor degeneration)	Once daily	Apoptosis, photoreceptor morphology, retinal functionality (ERG)	Retinal delivery demonstrated; quantitative retinal levels not reported	Preclinical
SOCS1-derived peptide (MIS1) [102]	JAK/STAT inhibitor	Reduces Müller cell activation and neuroinflammation	Experimental diabetes (STZ diabetic mice)	Twice daily	Müller cell activation, neuroinflammation, vascular leakage, gliosis, inflammatory cytokines, retinal thickness	Retinal delivery demonstrated; quantitative retinal levels not reported	Preclinical
Nerve growth factor (NGF) eyedrops [73,75]	Neurotrophic factor in nanocarrier	Supports neuronal survival	Corneal disease (clinical); experimental retinal degeneration/optic neuropathy models	Multiple daily instillations (typically 2–6/day depending on formulation)	Neuronal survival, retinal ganglion cell function, retinal morphology, visual function	Retinal penetration uncertain; no quantitative retinal PK reported	Phase I/II clinical trial
Erythropoietin-β nanoparticles (EPOβ) eyedrops [103]	Cytokine neuroprotective factor	Anti-apoptotic retinal protection	Healthy Wistar Hannover rats (ocular PK/biodistribution model)	Single-dose topical administration	Ocular biodistribution, tolerability, retinal exposure	Retinal/choroidal delivery demonstrated via nanoparticles; quantitative retinal concentrations limited/not standardized	Preclinical
Resveratrol nano-eyedrops [104]	Polyphenol antioxidant	Reduces oxidative stress	Experimental diabetic retinopathy (STZ-induced diabetic rats)/H_2_O_2_-induced retinal oxidative stress model	Once or twice daily	Oxidative stress markers, inflammatory cytokines, retinal morphology, apoptosis, retinal function	Improved ocular bioavailability reported; quantitative retinal levels not reported	Preclinical
Curcumin nano-eyedrops [105]	Polyphenol anti-inflammatory	Reduces retinal inflammation and oxidative stress	Experimental retinal inflammation and diabetic retinal injury models (STZ-induced diabetic rats)	Once or twice daily	Retinal inflammation, oxidative stress, apoptosis, retinal morphology, inflammatory mediators	Enhanced retinal delivery suggested; quantitative retinal levels not reported	Preclinical
Citicoline eyedrops [106,107]	Neuroprotective metabolic compound	Enhances membrane phospholipid synthesis and mitochondrial function	Experimental diabetes-induced retinal neurodegeneration (db/db mice)//Patients with glaucoma (exploratory neuroretinal evidence)	Twice to three times daily	Retinal functionality (ERG), neural conduction, retinal ganglion cell survival, apoptosis, retinal morphology	Retinal delivery demonstrated in liposomal formulation; quantitative retinal levels not reported	Phase II clinical trial
Coenzyme Q10 + Vitamin E eyedrops [108]	Mitochondrial antioxidant	Improves mitochondrial function and neuronal survival	Experimental diabetic retinopathy (db/db mice)	Twice daily	Retinal functionality (ERG), mitochondrial function, oxidative stress, retinal ganglion cell survival, apoptosis, retinal morphology	Retinal delivery demonstrated; quantitative retinal levels not reported	Preclinical
TAT-PACAP/TAT-VIP eyedrops [109]	Cell-penetrating peptide neuroprotectants	Enhances retinal penetration and anti-inflammatory signaling	Retinal ischemia and excitotoxicity animal models	Multiple daily instillations (typically twice/day)	Retinal morphology, cell death, anti-inflammatory signaling, retinal ganglion cell survival	Enhanced retinal penetration demonstrated via TAT conjugation; quantitative retinal levels not reported	Preclinical
BDNF nano-eyedrops (Brain-Derived Neurotrophic Factor) [110]	Neurotrophic factor	Promotes survival of retinal ganglion cells and synaptic plasticity	Light-induced retinal degeneration model	Once daily	Photoreceptor survival, retinal morphology, apoptosis, retinal functionality (ERG)	Retinal delivery demonstrated after conjunctival administration; quantitative retinal levels not reported	Preclinical
Ripasudil/Fasudil eyedrops (ROCK inhibitors) [111,112]	Small molecule (ROCK inhibitor)	Improves retinal blood flow, reduces neuroinflammation and apoptosis	Glaucoma/ocular hypertension models (trabecular meshwork dysfunction; pigmentary glaucoma ex vivo)	Once or twice daily	IOP, retinal blood flow, neuroinflammation, apoptosis, retinal ganglion cell protection	Posterior segment exposure suggested; quantitative retinal levels not reported	Approved (Japan) for glaucoma/OHT; Phase II/III clinical trials (glaucoma); exploratory (retina)

## Data Availability

No new data were created or analyzed in this study.

## Abbreviations

The following abbreviations are used in this manuscript:

AGEs	Advanced glycation end-products
BDNF	Brain-Derived Neurotrophic Factor
BRB	Blood-Retinal Barrier
DPP-4	Dipeptidyl Peptidase-4
DRD	Diabetic Retinal Disease
EPOβ	Erythropoietin beta
ERG	Electroretinography
GFAP	Glial Fibrillary Acidic Protein
GLP-1	Glucagon-Like Peptide-1
GLP-1R	Glucagon-Like Peptide-1 Receptor
GLP-1RAs	Glucagon-Like Peptide-1 Receptor Agonist(s)
IL-18	Interleukin 18
IL-1β	Interleukin-1 beta
iNOS	Inducible Nitric Oxide Synthase
IOP	Intraocular Pressure
IRBP	Interstitial Retinol-Binding Protein
JAK/STAT	Janus kinase/Signal Transducer and Activator of Transcription pathway
mfERG	Multifocal Electroretinography
MIS1	SOCS1-derived peptide
NF-κB	Nuclear Factor kappa B
NfL	Neurofilament Light Chain
NGF	Nerve Growth Factor
NLRP3	NOD-like receptor family pyrin domain-containing 3
NMDA	N-methyl-D-aspartate
nNOS	Neuronal Nitric Oxide Synthase
NO	Nitric Oxide
NVU	Neurovascular Unit
OCT	Optical Coherence Tomography
OCTA	Optical Coherence Tomography Angiography
PACAP	Pituitary Adenylate Cyclase-Activating Polypeptide
PEDF	Pigment Epithelium-Derived Factor
PSD-95	Postsynaptic Density Protein 95
RGC	Retinal Ganglion Cells
ROCK	Rho-associated protein kinase
ROS	Reactive Oxygen Species
SNAP25	Synaptosomal-Associated Protein 25
SOCS1	Suppressor of Cytokine Signaling 1
SST	Somatostatin
STZ	Streptozotocin
TNF-α	Tumor Necrosis Factor alpha
TRPV2	Transient Receptor Potential Vanilloid 2
VEGF	Vascular Endothelial Growth Factor
